# Correction: Hypoxia Preconditioned Mesenchymal Stem Cells Prevent Cardiac Fibroblast Activation and Collagen Production via Leptin

**DOI:** 10.1371/journal.pone.0143983

**Published:** 2015-12-03

**Authors:** Panpan Chen, Rongrong Wu, Wei Zhu, Zhi Jiang, Yinchuan Xu, Han Chen, Zhaocai Zhang, Huiqiang Chen, Ling Zhang, Hong Yu, Jian'an Wang, Xinyang Hu

The images of the β-actin bands in Fig 1C are erroneously reversed. In addition, the β-actin images in Fig 1C were mistakenly copied into Fig 1D. Please see the revised Fig 1 here, with corrected panels C and D, as well as correspondingly corrected panels B and E. The authors confirm that these errors do not change the conclusions of the paper and have provided the raw data for each affected panel as Supporting Information of this correction.

**Fig 1:**
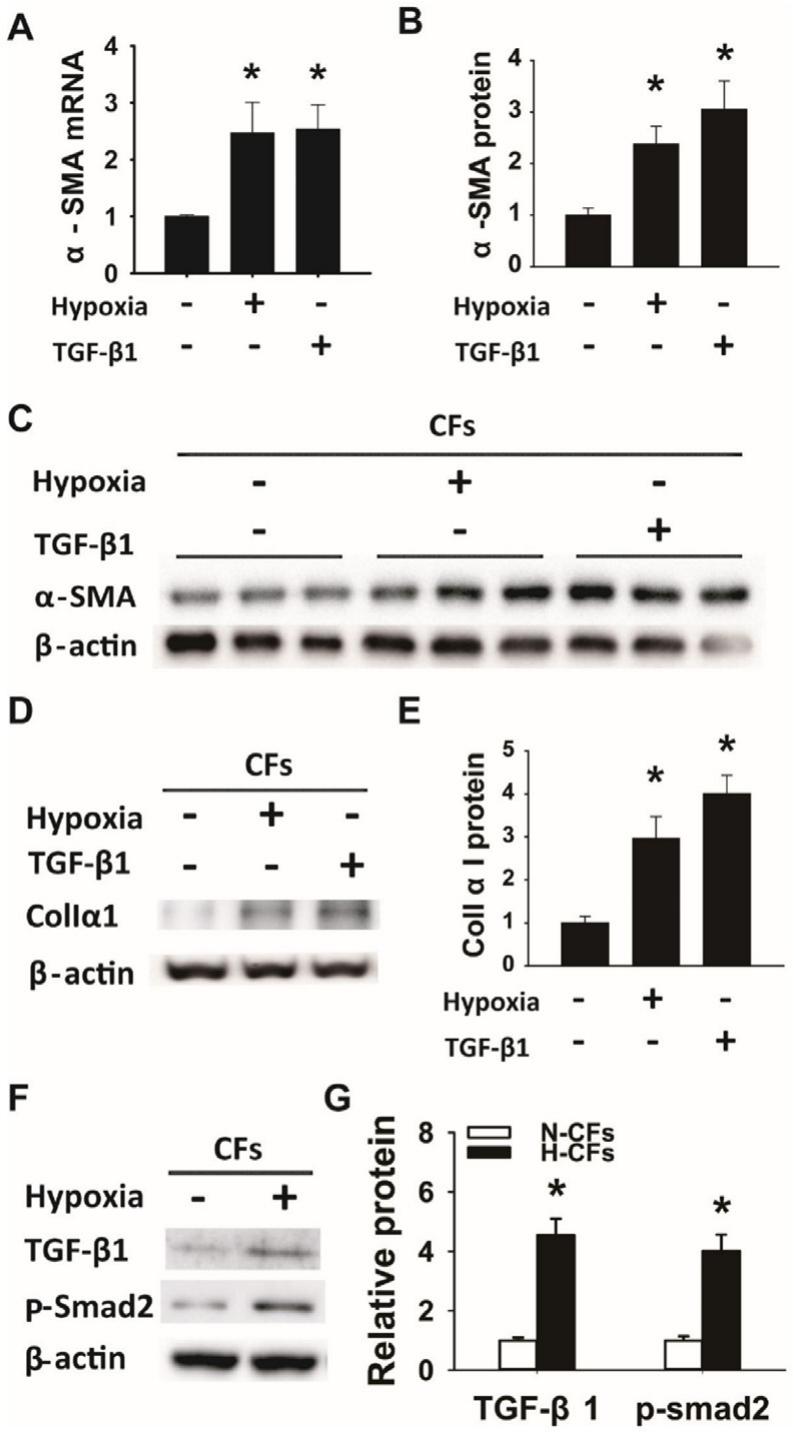
Hypoxia activated cardiac fibroblasts (CFs) to myofibroblasts (MCFs) through TGF-β/Smad2 pathway and increased collagen production. Cardiac fibroblasts cultured at normoxia (N-CFs) and hypoxia condition (H-CFs), or treated with 10 ng/ml TGF-β1 at normoxia condition 24 hours (N-CFs-TGF-β1) were collected for subsequent experiments. (A) α-SMA mRNA level measured by qRT-PCR of total RNA isolated from CFs. mRNA value of H-CFs and N-CFs-TGF-β1 was normalized to N-CFs. (B) Western blots quantification of α-SMA protein expression in cell lysates from CFs after hypoxia and TGF-β1 pretreatment. The α-SMA ratios normalized to β-actin in both hypoxia and TGF-β1 treated groups were compared with the control group. (C) Representative western blot analysis of α-SMA protein expression in CFs cultured under standard condition was used as a control. Both hypoxia and TGF-β1 treatment for 24 hours increased α-SMA expression. (D) Representative western blot of collagen IαI protein expression in cell lysates from N-CFs, H-CFs and N-CFs-TGF-β1. Both hypoxia and TGF-β1 treatment for 24 hours increased collagen IαI expression. (E) Quantification of collagen IαI western blot in D. The ratios of collagen IαI normalized to β-actin in both hypoxia and TGF-β1 pretreated groups were compared with the normoxia cultured group. (F) Representative western blot of TGF-β1 and phospho-Smad2 protein expression in N-CFs and H-CFs. (G) Quantification of TGF-β1 and phospho-Smad2 western blot in F. The ratio of TGF-β1 and p-Smad2 in H-CFs were compared to normoxia cultured CFs (n  =  3, *p<0.05).

## Supporting Information

S1 File Raw data for Fig 1. (PPTX)
