# Supplementary material for: Correction: Hypoxia Preconditioned Mesenchymal Stem Cells Prevent Cardiac Fibroblast Activation and Collagen Production via Leptin
Source: PLoS One. 2015 Dec 3;10(12):e0143983. doi: 10.1371/journal.pone.0143983 (PMC4669181; doi:10.1371/journal.pone.0143983)
Supplement: S1 File — (PPTX) [file pone.0143983.s001.pptx]

## Slide 1
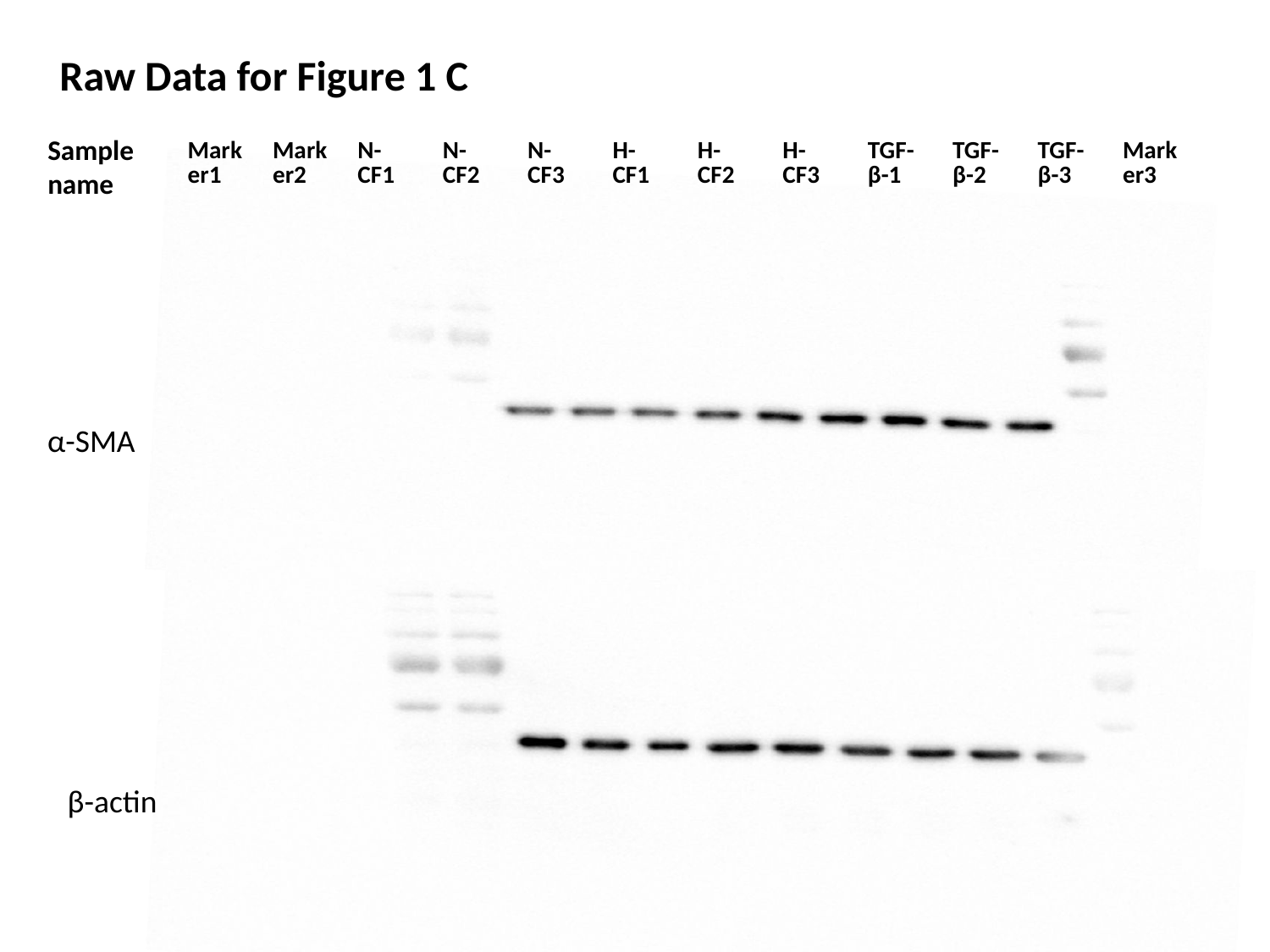

Raw Data for Figure 1 C
Sample
name
| Marker1 | Marker2 | N-CF1 | N-CF2 | N-CF3 | H-CF1 | H-CF2 | H-CF3 | TGF-β-1 | TGF-β-2 | TGF-β-3 | Marker3 |
| --- | --- | --- | --- | --- | --- | --- | --- | --- | --- | --- | --- |
α-SMA
β-actin

## Slide 2
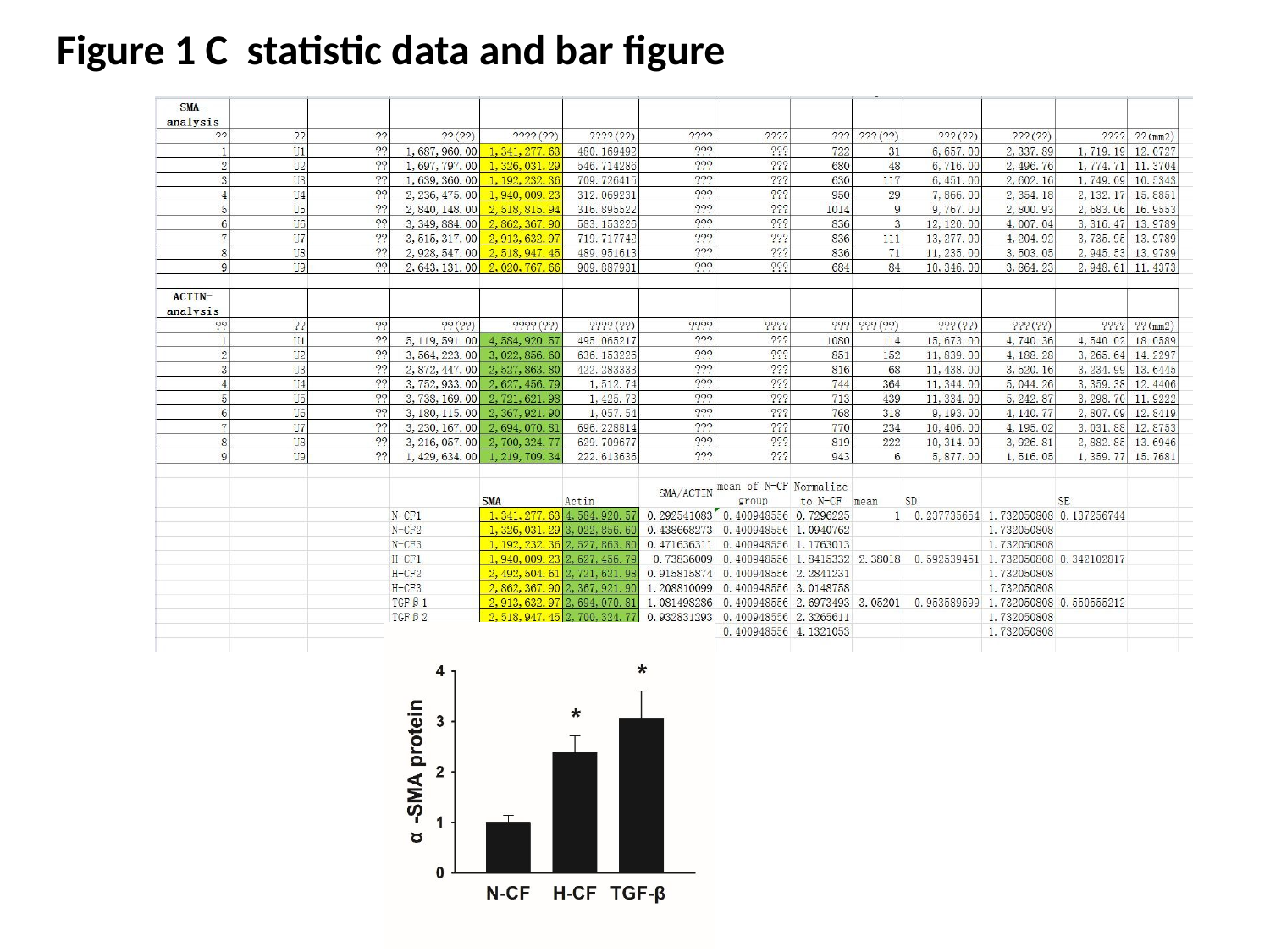

Figure 1 C statistic data and bar figure

## Slide 3
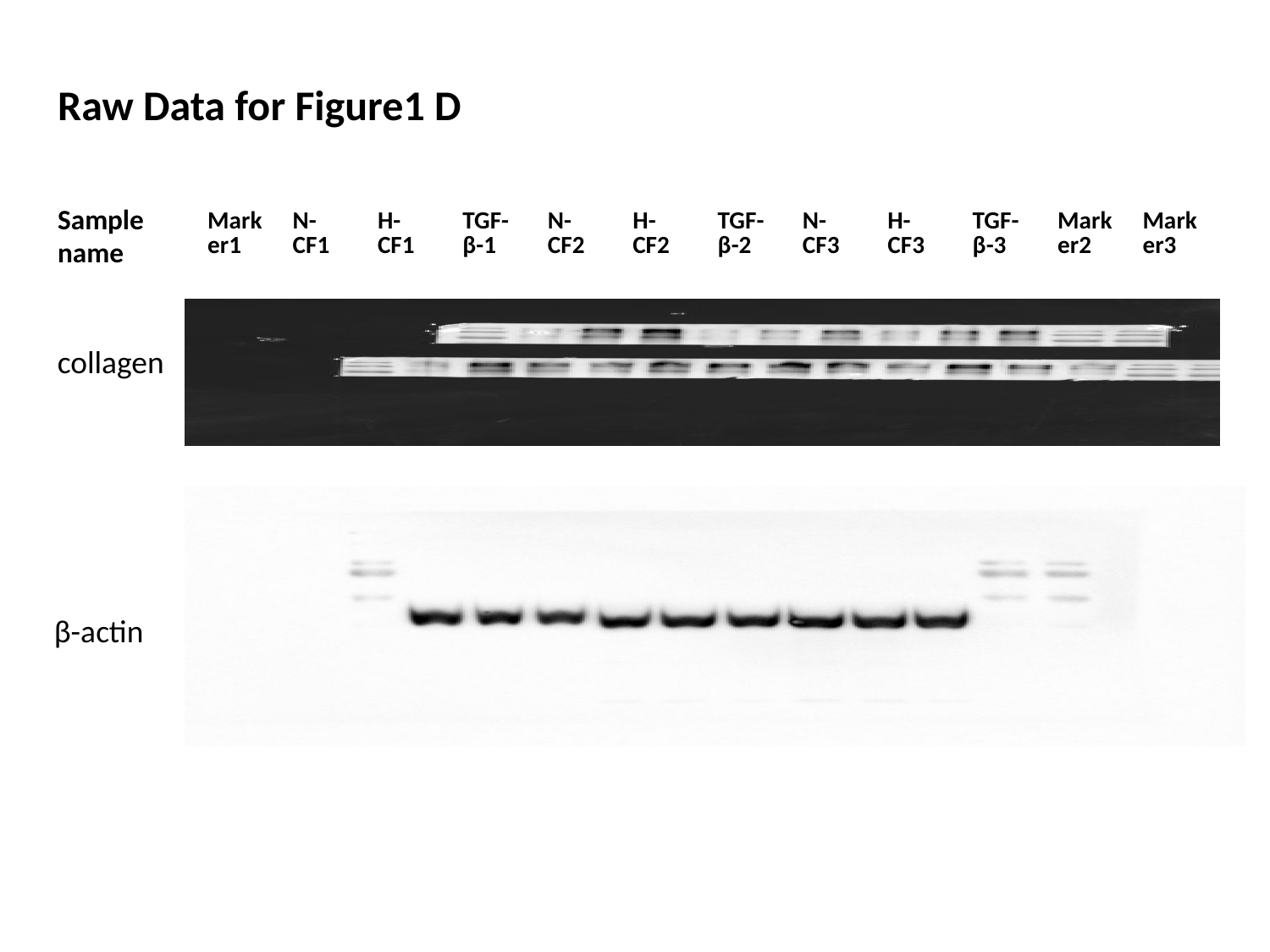

Raw Data for Figure1 D
Sample
name
| Marker1 | N-CF1 | H-CF1 | TGF-β-1 | N-CF2 | H-CF2 | TGF-β-2 | N-CF3 | H-CF3 | TGF-β-3 | Marker2 | Marker3 |
| --- | --- | --- | --- | --- | --- | --- | --- | --- | --- | --- | --- |
collagen
β-actin

## Slide 4
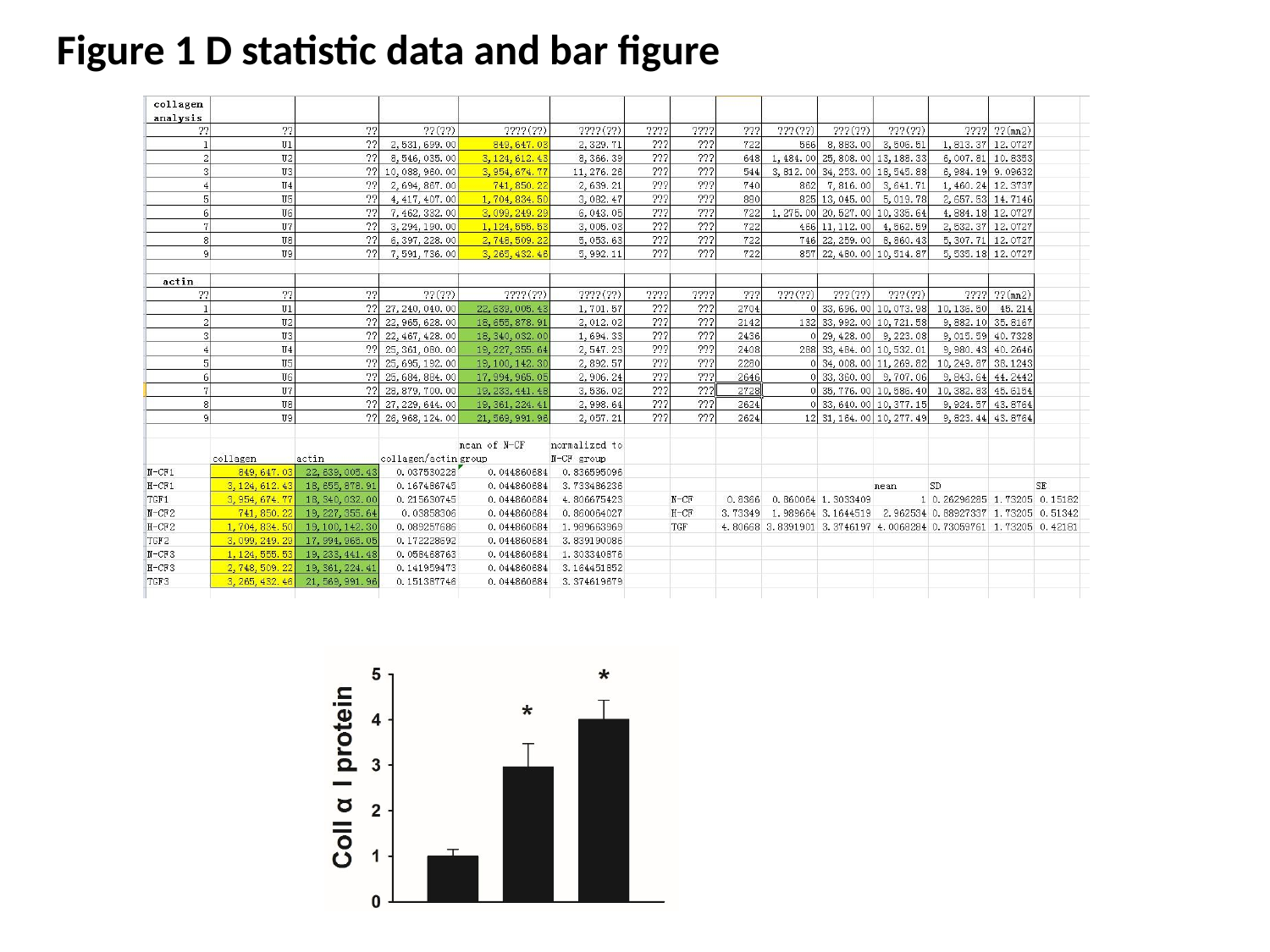

Figure 1 D statistic data and bar figure

## Slide 5
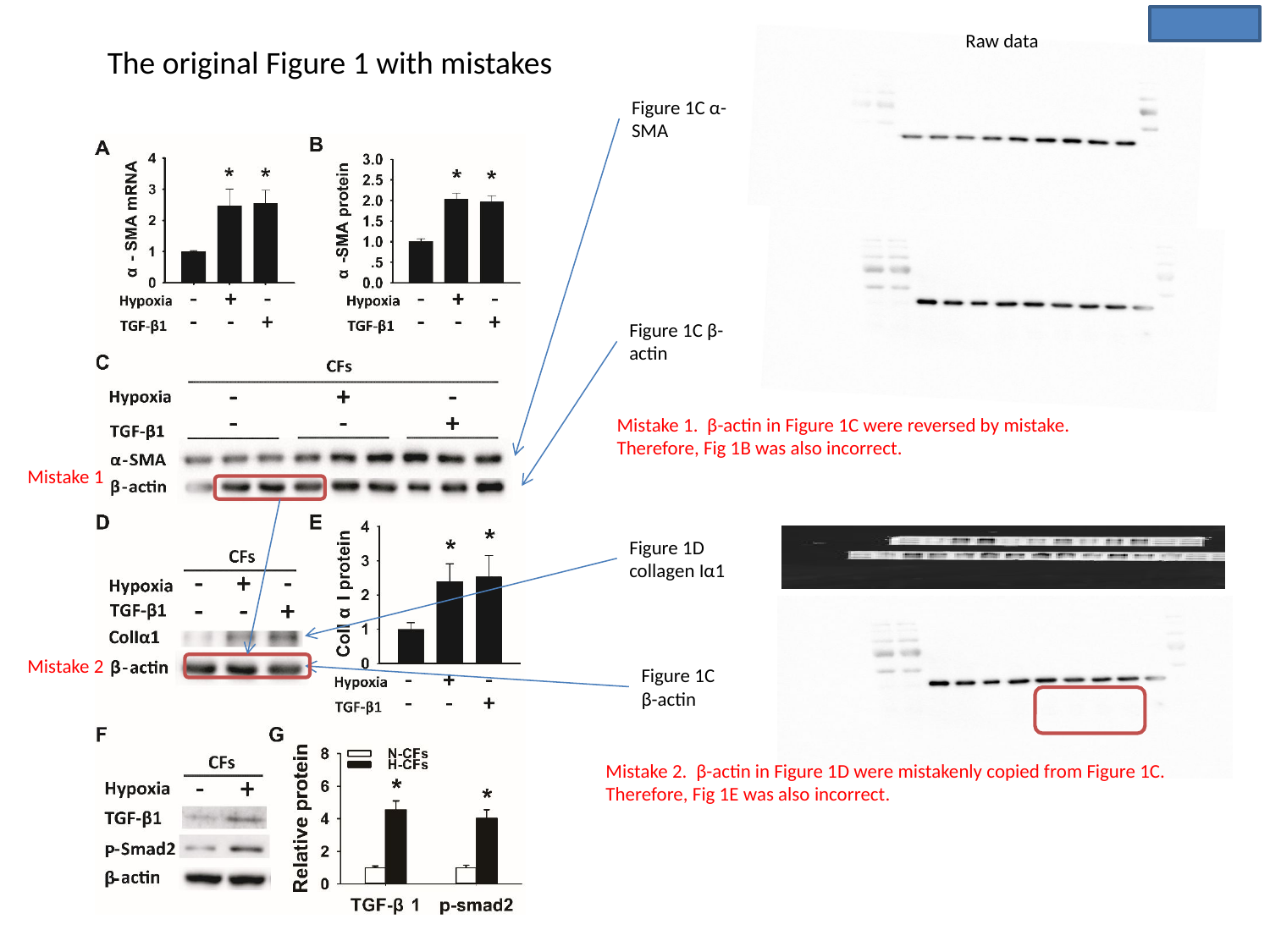

Raw data
The original Figure 1 with mistakes
Figure 1C α-SMA
Figure 1C β-actin
Mistake 1. β-actin in Figure 1C were reversed by mistake. Therefore, Fig 1B was also incorrect.
Mistake 1
Figure 1D collagen Iα1
Mistake 2
Figure 1C β-actin
Mistake 2. β-actin in Figure 1D were mistakenly copied from Figure 1C. Therefore, Fig 1E was also incorrect.

## Slide 6
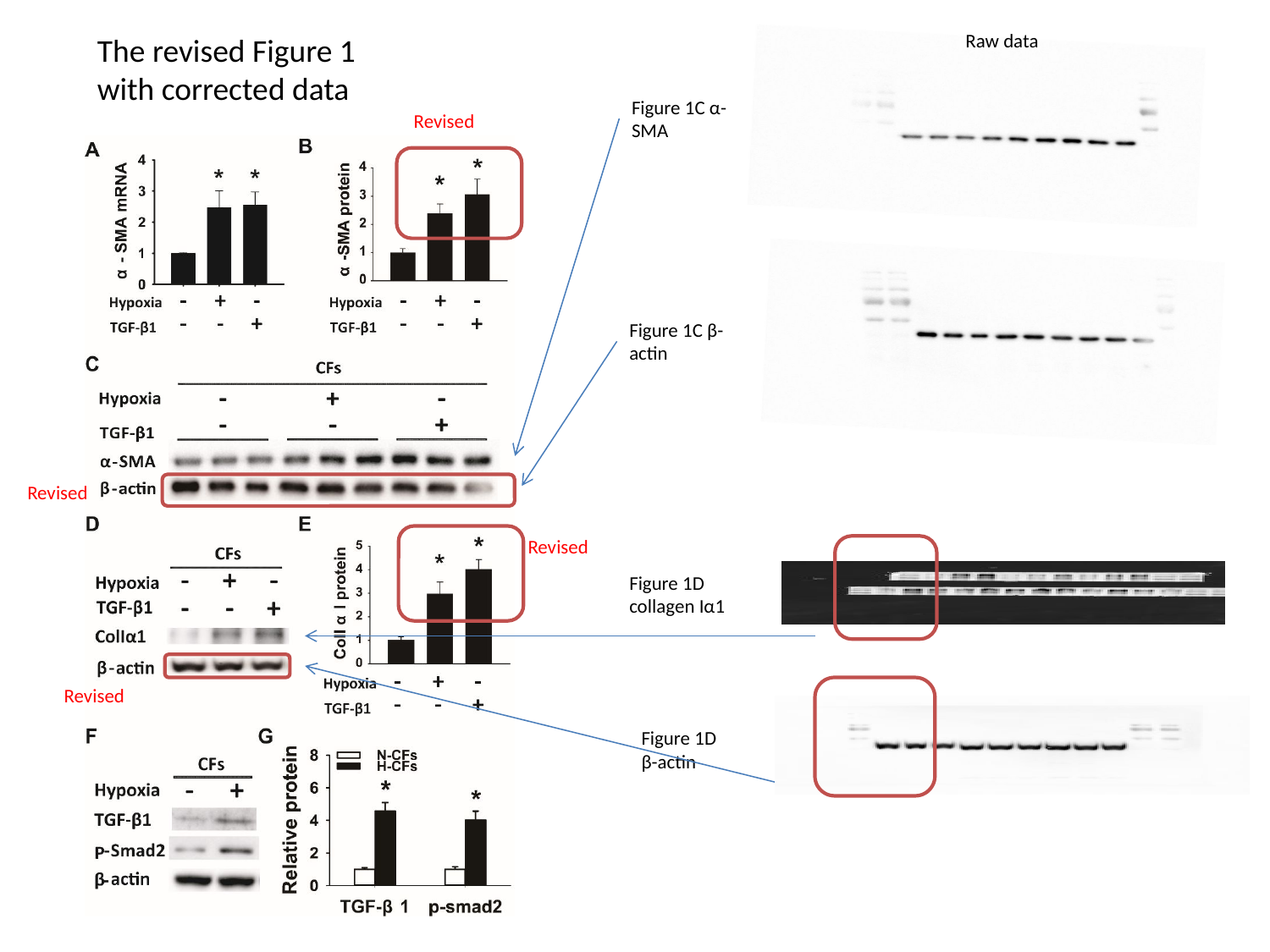

Raw data
The revised Figure 1 with corrected data
Figure 1C α-SMA
Revised
Figure 1C β-actin
Revised
Revised
Figure 1D collagen Iα1
Revised
Figure 1D β-actin
